# Single-lead electrocardiogram Artificial Intelligence model with risk factors detects atrial fibrillation during sinus rhythm

**DOI:** 10.1093/europace/euad354

**Published:** 2023-12-11

**Authors:** Stijn Dupulthys, Karl Dujardin, Wim Anné, Peter Pollet, Maarten Vanhaverbeke, David McAuliffe, Pieter-Jan Lammertyn, Louise Berteloot, Nathalie Mertens, Peter De Jaeger

**Affiliations:** RADar Learning and Innovation Centre, AZ Delta, Deltalaan 1, 8800 Roeselare, Belgium; Department of Cardiology, AZ Delta, Roeselare, Belgium; Department of Cardiology, AZ Delta, Roeselare, Belgium; Department of Cardiology, AZ Delta, Roeselare, Belgium; Department of Cardiology, AZ Delta, Roeselare, Belgium; Resero Limited, Dublin, Ireland; RADar Learning and Innovation Centre, AZ Delta, Deltalaan 1, 8800 Roeselare, Belgium; RADar Learning and Innovation Centre, AZ Delta, Roeselare, Belgium; RADar Learning and Innovation Centre, AZ Delta, Deltalaan 1, 8800 Roeselare, Belgium; RADar Learning and Innovation Centre, AZ Delta, Deltalaan 1, 8800 Roeselare, Belgium; Department of Medicine and Life Sciences, Hasselt University, Martelarenlaan 42, 3500 Hasselt, Belgium

**Keywords:** Atrial fibrillation, Single-lead ECG, Sinus rhythm, Artificial intelligence, Screening

## Abstract

**Aims:**

Guidelines recommend opportunistic screening for atrial fibrillation (AF), using a 30 s single-lead electrocardiogram (ECG) recorded by a wearable device. Since many patients have paroxysmal AF, identification of patients at high risk presenting with sinus rhythm (SR) may increase the yield of subsequent long-term cardiac monitoring. The aim is to evaluate an AI-algorithm trained on 10 s single-lead ECG with or without risk factors to predict AF.

**Methods and results:**

This retrospective study used 13 479 ECGs from AF patients in SR around the time of diagnosis and 53 916 age- and sex-matched control ECGs, augmented with 17 risk factors extracted from electronic health records. AI models were trained and compared using 1- or 12-lead ECGs, with or without risk factors. Model bias was evaluated by age- and sex-stratification of results. Random forest models identified the most relevant risk factors. The single-lead model achieved an area under the curve of 0.74, which increased to 0.76 by adding six risk factors (95% confidence interval: 0.74–0.79). This model matched the performance of a 12-lead model. Results are stable for both sexes, over ages ranging from 40 to 90 years. Out of 17 clinical variables, 6 were sufficient for optimal accuracy of the model: hypertension, heart failure, valvular disease, history of myocardial infarction, age, and sex.

**Conclusion:**

An AI model using a single-lead SR ECG and six risk factors can identify patients with concurrent AF with similar accuracy as a 12-lead ECG-AI model. An age- and sex-matched data set leads to an unbiased model with consistent predictions across age groups.

## Graphical Abstract

**Graphical Abstract:**
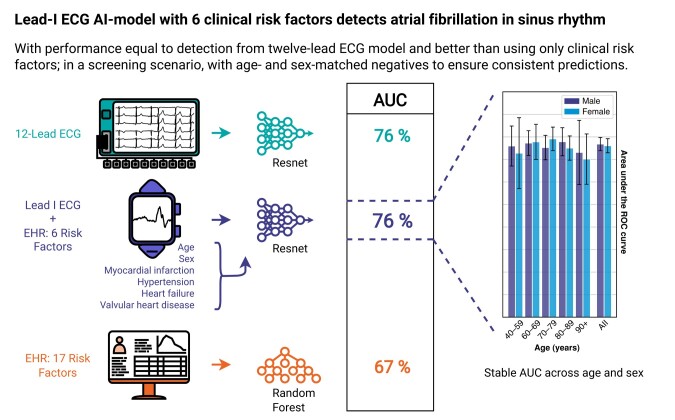
AUC, area under the receiver operating characteristic curve; EHR, electronic health records

**What’s new?**

- Can an AI-enhanced 10 s single-lead electrocardiogram (ECG) with electronic health record (EHR)-extracted risk factors be used to identify subclinical atrial fibrillation (AF) during sinus rhythm (SR) in a screening scenario?
- An AI-enhanced single-lead ECG with six cardiovascular risk factors performs equally well as a 12-lead ECG in a retrospective dataset of patients with AF and age- and sex-matched controls; significantly outperforming a pure risk factor–based classification. Performance is stable over age- and sex-stratification, allowing for reliable patient-level predictions.
- Atrial fibrillation detection is possible using an AI-enhanced single-lead ECG during SR in a screening scenario. Augmented with EHR-extracted cardiovascular risk factors, the Lead-I ECG model reaches the performance of an AI-enhanced 12-lead ECG.

## Introduction

Atrial fibrillation and flutter (AF) are the most common arrhythmias, with an estimated prevalence of 60 million cases worldwide^1^ and prevalence rates around 8% in people above 55 years of age.^2^ It is a significant risk factor for ischaemic stroke, the number two most common cause of death worldwide.^1^ Approximately 13% of AF cases go undiagnosed.^3^ One-third of all known AF cases present without symptoms;^4^ this is most commonly paroxysmal AF and has similar outcomes as symptomatic AF.^5^

The life-time risk of AF is determined by age, sex, genetic, and (sub)clinical risk factors.^6–8^ The CHARGE-AF score^9^ is often used to estimate the probability of diagnosing AF in the next 5 years and the CHA_2_DS_2_-VASc-score^10^ is used to classify the risk of stroke and thromboembolic events.

European and US guidelines recommend opportunistic or systematic screening for AF with pulse taking or single-lead electrocardiogram (ECG) in individuals above a certain age or who are at risk for stroke.^11^ The challenge is to identify patients who have undiagnosed intermittent AF but who are in sinus rhythm (SR) at the time of screening, in whom prolonged ambulatory cardiac rhythm monitoring using implantable loop recorders or wearables is advisable. However, these strategies are expensive, invasive, or inconvenient, require a home monitoring set-up, have a low diagnostic yield, and may also detect patients with a low burden of AF that is of uncertain clinical significance.^12^

New wearable ECG-monitoring devices and the use of AI in electrocardiography are transforming the field of electrophysiology.^13^ An AI-enhanced algorithm applied to ECG during SR has recently been shown to detect concurrent, episodic clinical AF^14,15^ and allows for separating high- from low-risk patients for more efficient use of cardiac monitoring.^16^ It is however unclear whether the performance of a neural network trained on ECGs in different study populations could be affected by imbalances in gender, age, and (sub)clinical risk factors for stroke. Furthermore, it is unclear whether AI ECG detection of AF could be further improved by incorporating clinical risk factors predictive of incident AF. Finally, we aimed to evaluate whether a neural network performed equally well when trained using 1- or 12-lead ECG, with or without risk factors.

## Methods

### Study population and data sources

This retrospective study used data collected from the ECG and electronic health record (EHR) databases from AZ Delta (Roeselare, Belgium), from all departments. The ECG dataset contains 173 537 ECGs, from 68 880 patients, recorded between 13 July 2004 and 30 April 2022 and stored in the GE MUSE Cardiology Information System. All ECGs are 10 s, acquired at a sampling rate of 500 Hz. Diagnostic labels are assigned by the MUSE system. Structured clinical data comes from the hospital’s APR-DRG system as ICD9 and ICD10 diagnostic and procedural codes, and from the HiX EHR system (Chipsoft) as physician diagnoses, drug prescriptions, and measurements. The structured data, including ECG diagnostic labels, were transformed into the Observational Medical Outcomes Partnership (OMOP) common data model (v5.4) developed by the Observational Health Data Sciences and Informatics collaborative. The OMOP dataset is built using the Rabbit-In-A-Blender pipeline^17^ in BigQuery on the Google Cloud Platform. The OMOP implementation is supported by a grant from the European Health Data Evidence Network (EHDEN); this study has support from Flanders Innovation & Entrepreneurship (VLAIO) through the Advanced Data-Aided Medicine (ADAM) project. This study was approved by the hospital’s ethical committee as part of the ADAM project.

#### Electrocardiogram selection

The ECG data set contains ECGs that meet the selection criteria for either the positive or negative case group. The positive case group contains SR–ECGs from patients with confirmed AF, i.e. with at least one ECG with an AF diagnosis. AF–ECGs occurring within 7 days after coronary artery bypass graft (CABG) surgery or valve procedures were excluded as they can be triggered by the procedure rather than signify established AF. Sinus rhythm–ECGs were included starting from 91 days before the first AF–ECG. Sinus rhythm–ECGs after a set of (invasive) treatments are excluded, more specifically after left atrial appendage closure, ablation therapy, cardioversion, pacemaker/implantable cardioverter-defibrillator insertion, and heart transplant. The negative case group contains all SR–ECGs from patients who never had a diagnosis of AF. In order to limit the number of false negatives, patients are also filtered out if they have any history of (possible) AF-related treatments (left atrial appendage closure, ablation therapy, cardioversion, pacemaker/implantable cardioverter-defibrillator insertion, use of oral anticoagulants (OAC), use of antiarrhythmic drugs), or have a (likely positive) mention of AF in their medical notes. Patients with heart transplants are excluded as well. The selection criteria for ECGs are summarized in Supplementary material online, *Table S1*. Observational Medical Outcomes Partnership concept-ids for the concepts used in these criteria are provided in Supplementary material online, *File S1*.

#### Clinical risk factors

Age, sex, obesity, and (any history of) AF risk factors are extracted from the OMOP database relative to the date of each SR–ECG. The risk factor had to be registered before recording the SR–ECG; no other time, number of repeated diagnoses, or treatment type constraints are imposed. The choice of risk factors is a simplified subset of risk factors found in the CHA_2_DS_2_-VASc-score,^10^ the CHARGE-AF score,^9^ and the EHR-AF score,^18^ depending on the granularity and accuracy of the available data. An overview of included risk factors is shown in *Table 1*. Concept-ids used for these risk factors are based on ICD-9 and ICD-10 codes from the EHR-AF score^18^ and the algorithm for the Elixhauser Comorbidity Measure from Quan *et al.*,^19^ mapped to OMOP’s standard concept-ids and supplemented with other relevant concept-ids; these concept-ids are available in Supplementary material online, *File S2*.

**Table 1:** Clinical risk factors assessed relative to the date of each SR ECG

**Patient characteristics**
Age (numeric)
Obesity or BMI ≥30
Sex
Smoking (any exposure)
**Medical history**
Coronary artery bypass graft present
Chronic kidney disease
Chronic obstructive pulmonary disease
Diabetes mellitus
Heart failure
Heart valve disease or valve procedure
Hypertension
Hyperthyroidism
Hypothyroidism
Myocardial infarction
Obstructive sleep apnoea syndrome
Peripheral artery disease
Stroke or transient ischaemic attack

All parameters are binary, except for age in years.

BMI, body mass index.

### AI model

#### Datasets

The ECG records and clinical risk factors are combined, and the individual records are divided into a train, validation, and test set in an 80–10–10% split by the patient. Two subsets are constructed; the first dataset, termed the matched dataset, comprises AF patients and an age- and sex-matched control population. The second, termed the replication dataset, follows the approach of a previous study^14^ for literature comparison. Age-filtering and subset selection are done in *Python* (3.8.10), using the *PyArrow* (11.0.0) and *Polars* (0.16.14) packages.

The matched dataset contains ECGs taken at age 40 years or older. Only SR–ECGs in a window between 91 days before and 365 days after the first AF–ECG are kept for the positive cases. For the test and validation set, this is limited to the first SR–ECG per patient in the window. The negative case group contains four sex- and age-matched SR–ECGs per positive case SR–ECG. These ECGs are selected at random from all SR–ECGs from the negative cases in the respective set, but limited to one ECG per patient for the validation and test sets.

The replication dataset is filtered for ECGs taken at age 18 or over. Sinus rhythm–ECGs for positive cases start 31 days before the first AF–ECG. No other filtering is done on the included positive and negative cases for the training set. The validation and test sets are reduced to one ECG per patient: in the positive case group, only the first SR–ECG in the 31 days leading up to the first AF–ECG is kept; for the negative case group, the first SR–ECG overall is kept.

#### Model architecture

**Figure 1:**
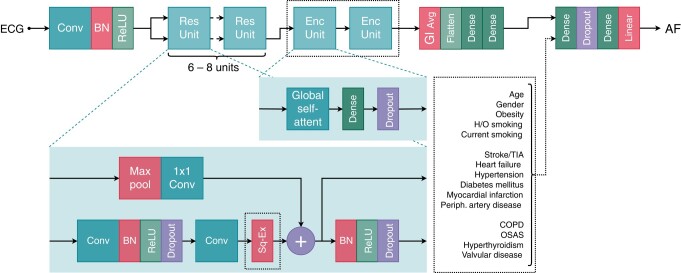
Residual neural network architecture. Dotted boxes show model variations: squeeze-excite layers in residual units, encoder-layers, additional structured data input. Input either single-lead or 12-lead ECG, output as logit values, convertible to probabilities using the logistic function. AF, atrial fibrillation; BN, batch normalization layer; Conv, convolution layer; COPD, chronic obstructive pulmonary disease; Enc Unit, encoding unit; GlAvg, global averaging layer; H/O, history of; OSAS, obstructive sleep apnoea syndrome; ReLU, rectified linear unit; Res Unit, residual unit; Self-Attent, self-attention; Sq-Ex, squeeze-excite layer; TIA, transient ischaemic attack.

Multiple residual neural network (ResNet) architectures are evaluated, similar to previous studies,^14,20,21^ but modified to use 1 (Lead I) or 12 ECG leads and optional side-input of clinical risk factors. The models follow the template shown in *Figure 1*; more details can be found in Supplementary material online, Tables S2 and*S3*; the model code is in Supplementary material online, *File S3*.

Two random forest (RF) classifiers are fitted for comparison. The first uses only the clinical risk factors as input. The second uses both the clinical risk factors and a feature vector of the ECG. This feature vector contains the activations from the second to last Dense layer of the 12-lead ECG model, which lies in a space optimized for the classification of positive and negative cases. Details on the model hyperparameters can be found in Supplementary material online, *Table S4*.

All model training and evaluation are done on a local workstation with an Intel Xeon Silver 4214R CPU, 256 GB of memory, and an NVIDIA RTX A6000 GPU, in a containerized environment. The ResNets are implemented in *TensorFlow* (2.11.1) using the Keras API; the RFs are trained using *scikit-learn* (1.2.2).

### Outcomes of interest

The outcome of interest is the diagnostic performance of the best single-lead ECG model with or without additional risk factors, compared with the best 12-lead ECG-only model for the matched dataset. The diagnostic performance of a model is defined as the area under the curve (AUC) of the receiver operating characteristic (ROC) curves for the test set. The relevance of individual clinical risk factors is assessed by an RF classifier trained on the risk factors. A single-lead ECG model with reduced risk factors is evaluated using the most relevant risk factors. Model performance is stratified by age and sex to study the stability over varying age and sex groups for the single-lead ECG model with reduced risk factors.

As a secondary analysis, the diagnostic performance of the models trained using the replication dataset is compared with the literature. Model bias is assessed using correlations between clinical risk factors and an ECG feature vector extracted from the trained model. Stability over age and sex groups is evaluated by stratification of results by age and sex.

### Statistical analysis

The outcome of interest depends on the AUC of the ROC curve for different models. This AUC is calculated using the *roc-curve* and *auc* methods from *scikit-learn*, on the true and predicted labels. Confidence intervals (CIs) for these AUCs are calculated using a bootstrapping method with resampling implemented in Python. Area under the curves stratified for age and sex are used to assess bias. The relative importance of individual risk factors in the RF classifier is assessed using Shapley Additive Explanations (SHAP) values, as calculated by the *shap* package.^22,23^ Point-biserial correlation coefficient between the ECG feature vector and the risk factors is calculated using *pointbiserialr* from *scipy*.

## Results

### Study population, datasets, and model selection

**Figure 2:**
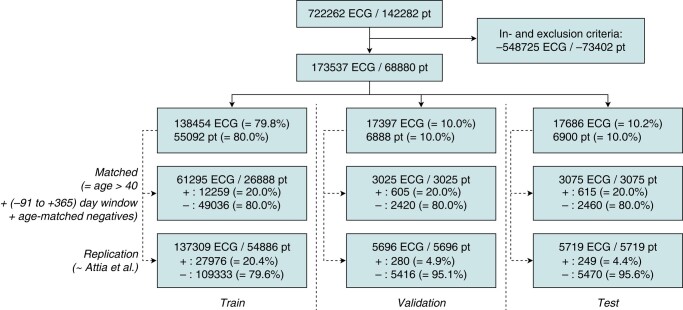
ECG dataset with train-validation-test split for replication and matched datasets. ECGs are split in an 80–10–10% split by the patient. The replication dataset uses a time window starting 31 days before the first AF–ECG for positive cases, and selects ECGs for the validation and test sets as in Attia *et al*.^14^ The matched dataset filters for ages above 40 years, uses a time window of 91 days before up to 365 days after the first AF–ECG for positive cases, and uses a 4-to-1 age-matching approach for the negative cases. ECG, electrocardiogram; pt, patients; +, positive cases; −, negative cases.

The ECG dataset is filtered using the (general) in- and exclusion criteria shown in *Table 1* and split into a training, validation, and test set. These sets are processed to match a replication dataset, as shown in *Figure 2*. The sets are compared for age, sex, and (estimated) CHA_2_DS_2_-VASc-score in *Tables 2* and *3* and for clinical risk factors in Supplementary material online, *Tables S5* and *S6*, for the matched and replication datasets, respectively. Supplementary material online, *Figure S1* shows the performance for varying model architectures and inputs; performance depends mostly on the input data, with limited benefit for larger, more complex, models, so only the deep_resnet_encoder-architecture is evaluated below.

**Table 2:** Study population characteristics for the matched dataset shows limited differences by selecting only age 40 years and above, by using a smaller time window around the first AF–ECG and especially by age- (and sex)-matching the negative cases

	+ (train)	−⁠ (train)	+ (val/test)	−⁠ (val/test)
Age (years)	75 (67–82)	73 (66–81)	75 (68–83)	74 (66–82)
Sex (male)	60.76%	60.76%	58.44%	58.44%
CHA_2_DS_2_-VASc-score	3 (2–4)	3 (1–4)	3 (2–4)	2 (1–3)

The remaining differences between positive and negative cases should mainly be related to the AF risk. Age and CHA_2_DS_2_-VASc-score as ‘median (first quartile-third quartile)’. +, positive case; −, negative case; val, validation

**Table 3:** Study population characteristics for replication dataset showing stark differences for negative cases between the training and validation/test set

	+ (train)	−⁠ (train)	+ (val/test)	−⁠ (val/test)
Age (years)	75 (67–82)	62 (50–74)	76 (68–83)	57 (44–70)
Sex (male)	60.76%	46.26%	58.44%	46.05%
CHA_2_DS_2_-VASc-score	4 (2–5)	2 (1–3)	3 (2–5)	1 (0–2)

Differences between positive and negative cases are related to AF risk, but exaggerated by age-bias in ECG-selection strategies. Age and CHA_2_DS_2_-VASc-score as ‘median (first quartile-third quartile)’.

+, positive case; −, negative case; val, validation.

### Model performance and stability for the matched dataset

*Table 4* shows the AUC per input data type, ranging from 0.74 to 0.78 for the test set. Both adding risk factors and increasing from 1 to 12 leads consistently improve AUC by 0.02. The performance of the model using Lead-I ECG and clinical risk factors matches the performance of the 12-lead ECG model. The performance of the 12-lead ECG model is in line with results from literature for age-matched ECGs.^21^

**Table 4:** Area under the ROC curve for AI models trained and evaluated on the matched and replication datasets, with mean and 95% CI, for varying inputs

Train	Test	Lead I	Lead-I + CRF	Lead-I + ORF	12-Lead	12-Lead + CRF
Match.	Match.	0.74 (0.72–0.76)	**0.76** (0.74–0.79)	**0.76** (0.74–0.79)	**0.76** (0.74–0.79)	0.78 (0.76–0.80)
Match.	Repli.	0.78 (0.75–0.82)	0.72 (0.69–0.76)	—	0.81 (0.78–0.84)	0.75 (0.72–0.79)
Repli.	Match.	0.74 (0.72–0.76)	0.73 (0.71–0.76)	—	0.76 (0.74–0.78)	0.76 (0.74–0.78)
Repli.	Repli.	0.83 (0.80–0.85)	0.88 (0.85–0.90)	—	0.84 (0.81–0.86)	0.89 (0.87–0.91)

Performance is equal between Lead-I ECG with full and reduced set of risk factors and 12-lead ECG models (bold values) and stable over different test sets for models trained on the matched dataset. Performance for models trained on the replication dataset is very sensitive to the specific test dataset, and the effect of adding risk factors is not reliable, due to bias in dataset construction.

CRF, clinical risk factors; ORF, optimal set or clinical risk factors.

**Figure 3:**
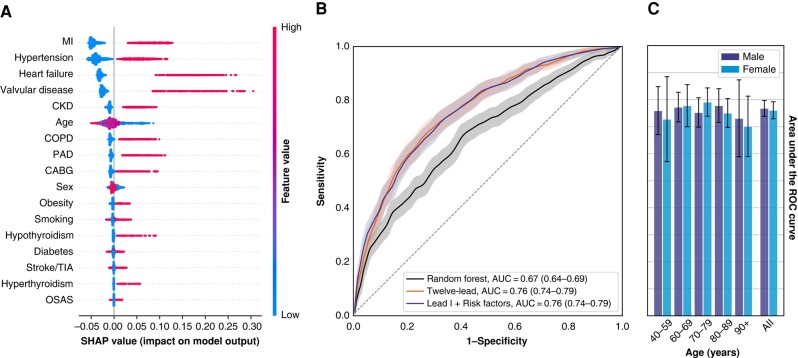
(*A*) Relative contributions of risk factors for the RF classifier. Four cardiovascular diseases are the most important. SHAP-values calculated for the matched dataset. (*B*) ROC curves for the single-lead ECG model with an optimal set of risk factors compared with full 12-lead ECG model and RF model using all risk factors. (*C*) Age- and sex-stratified AUC for single-lead ECG model with optimal risk factors, showing stable results over a large range of ages and both sexes. AUC, area under the curve; CABG, coronary artery bypass graft; CKD, chronic kidney disease; COPD, chronic obstructive pulmonary disease; MI, myocardial infarction; OSAS, obstructive sleep apnoea syndrome; PAD, peripheral artery disease; RF, random forest; TIA, transient ischaemic attack.

The RF-model, trained on the matched dataset and using only risk factors, has an AUC of 0.67 (*Table 5*). The four most important (cardiovascular) conditions for the classification are myocardial infarction, hypertension, heart failure, and valvular disease (*Figure 3A*). The inclusion of an ECG feature vector increases the AUC to 0.78, matching the AI model performance and demonstrating the benefit of adding ECG-derived information. Next to the ECG features, the same four cardiovascular diseases are important for this classification, see Supplementary material online, *Figure S2*. Retraining the single-lead ECG model with this optimal set of risk factors (four cardiovascular diseases, plus age, and sex) performs equally well as both the single-lead ECG model with all risk factors and the 12-lead ECG model (*Table 4* and *Figure 3B*).

**Table 5:** Area under the ROC curve for RF models trained and evaluated on the matched and replication datasets, with mean and 95% CI using risk factors and an ECG feature vector extracted from the 12-lead ECG model

Train	Test	Risk factors	Risk factors + ECG embedding vector
Match.	Match.	**0.67** (0.64–0.69)	**0.78** (0.76–0.80)
Match.	Repli.	0.73 (0.69–0.78)	0.79 (0.76–0.82)
Repli.	Match.	0.65 (0.63–0.68)	0.77 (0.75–0.79)
Repli.	Repli.	**0.86** (0.84–0.88)	**0.86** (0.84–0.89)

The addition of the ECG embedding vector significantly improves the performance of the classifier, almost reaching the original best AI model performance, for the matched dataset (bold values, top row). Models trained on the replication dataset have very high performance, while not benefiting from the addition of ECG features for the replication test set; this suggests possible bias in the risk factors, driving the decision (bold values, bottom row).

*Figure 3C* shows age- and sex-stratified AUCs for the single-lead ECG model with an optimal set of clinical risk factors for the matched dataset. Differences over varying age and sex are not significant. The results are stable, with no sex-related bias. Optimal performance is found for patients between 60 and 89 years, likely related to the larger amount of available training data.

### Model performance and bias for the replication dataset

*Table 4* contains the results for models trained on the replication dataset. The addition of risk factors increases the AUC by 0.05 for both the single-lead and 12-lead ECG models. When evaluating these models on the test set with matched cases, the model performance is lower and the addition of risk factors shows no benefit.

**Figure 4:**
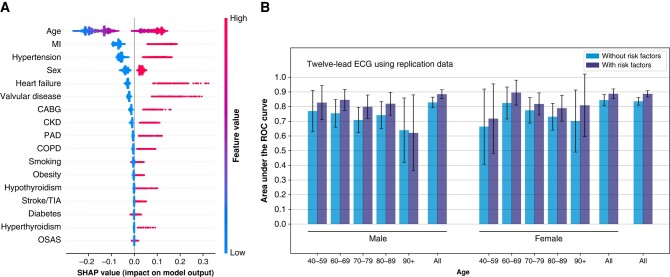
(*A*) Relative contributions of risk factors for the RF classifier in the replication analysis, using SHAP-values. Age is the dominant risk factor in the classification. (*B*) Age- and sex-stratified AUC for 12-lead ECG model with and without (all) risk factors. The age-stratified results show large differences depending on the inclusion of risk factors. Results are much more variable over age groups, compared with the models trained on the matched dataset. CABG, coronary artery bypass graft; CKD, chronic kidney disease; COPD, chronic obstructive pulmonary disease; MI, myocardial infarction; OSAS, obstructive sleap apnea syndrome; PAD, peripheral artery disease; TIA, transient ischaemic attack.

The RF-model, trained on the replication dataset and using only risk factors, has an AUC of 0.86. This is almost as good as the best AI models and does not benefit from adding information extracted from the ECG models (*Table 5*). Furthermore, the performance depends strongly on the ECG-selection criteria, as the AUC drops to 0.65 when evaluating the test set with matched cases. Age is the strongest driver for classification (*Figure 4A*), followed by cardiovascular risk factors and sex. Supplementary material online, *Figure S3A* shows the correlations between ECG features derived from the pure 12-lead ECG model and the risk factors; especially high correlations are seen for age and some cardiovascular risk factors.

Age- and sex-stratified results, as shown in *Figure 4B*, show a striking decrease in performance for the age-stratified results compared with the overall results, especially for the model without risk factors. The results are highly dependent on the inclusion of risk factors and vary a lot more over age groups than for the matched dataset.

## Discussion

Electrocardiogram screening for AF in patients in SR can improve screening efficiency by finding patients with a higher likelihood of having paroxysmal AF. We identified an age-independent, single-lead ECG model that reliably predicts AF when patients are in SR. Previous studies have suffered from age-related biases^24,25^ and were based on a 12-lead ECG. These findings may help to translate this approach to point-of-care devices in an outpatient setting or wearable devices.

Furthermore, we identified clinical risk factors, allowing a single-lead ECG model to perform as well as a full 12-lead model. Simplifying the number of risk factors to age, sex, hypertension, heart failure, history of myocardial infarction, and heart valve procedures performs equally well and might be better adapted to clinical practice. The next step towards integration in practice would be a prospective clinical trial to validate this algorithm in a screening setting in the outpatient cardiology clinic.

### Implications for AF screening

Single-lead ECG from a wearable ECG monitor can be used for an opportunistic, cost-effective screening program for AF.^26,27^ This strategy can be improved by identifying high-risk patients from ECGs in SR using an AI model. This idea was proven successful for 12-lead ECG;^16^ evidence for single-lead ECG models is sparse.

We developed single-lead ECG models, trained on age- and sex-matched patients and controls to approximate a screening scenario and allow for unbiased risk estimation for individual patients. Using only single-lead ECG, the best model achieved an AUC of 0.74; the addition of the six most important risk factors increased performance to an AUC of 0.76, matching the performance of the full 12-lead ECG model. Age- and sex-stratified results show stable performance and a small, but consistent benefit from the addition of risk factors (Supplementary material online, *Figure S4*).

Hygrell *et al*.^25^ developed a single-lead ECG–AI model using data from wearable ECG monitors from prospective AF screening studies. Their model achieved an AUC of 0.80 on the test data from the SAFER Feasibility Study for patients aged 65 years and above. However, the performance dropped to an AUC of 0.62 for the age-homogenous STROKESTOP I^28,29^ and STROKESTOP II^30,31^ studies, containing only 75- to 76-year-old patients. The performance of our single-lead ECG models for the subgroup of 75- to 76-year-old patients is more stable, with an AUC of 0.74 for the model with six clinical risk factors and 0.72 for the model without risk factors.

We highlight two important limitations to the application of this method in clinical practice. First, the relation with AF-burden was not assessed. This method can suggest a potential benefit of a more thorough diagnostic assessment, but makes no claims surrounding the severity of the AF or the effect of treatment. Second, current guidelines^11^ suggest using 30 s of single-lead ECG recordings, while 10 s was used in this study due to data availability. Its effect on prediction accuracy and over- or underdiagnosis is unknown and unpredictable.

### Model bias

Age- and sex-matching of the negative controls to the positive cases minimizes the bias in the dataset, resulting in age- and sex-stable models. An unbalanced dataset could lead to a biased model, as ECGs contain information on both age and sex,^32^ and there are important age- and sex-related differences in clinical risk factors. Case matching limits the differences in CHA_2_DS_2_-VASc-score (*Table 2*) and in clinical risk factors (Supplementary material online, *Table S5*), between the positive and negative cases. The remaining differences are likely related to the presence of AF, allowing for improved classification when risk factors are included in the model. Slight differences are present between the train and validation/test set, due to the inclusion of all SR–ECGs in the time window for the training set. Sicker patients are overrepresented in the training set due to a more intensive follow-up (and thus more ECGs) and more hospital admissions leading to a more complete structured record of their medical history (higher, more accurate risk factor rates). No race or other biases could be assessed due to limitations in the dataset.

In contrast, the ECG-selection strategy replicated from literature^14,15^ resulted in an important bias in age, where negative cases were recorded on average in significantly younger patients than positive cases, with a mean difference of 13.3 years in the test set.^14^ As age can quite reliably be predicted from ECGs,^32^ this overrepresentation of negative, younger and positive, older patients leads to a biased model. This presents itself as an increasing sensitivity and a decreasing specificity with age for their model.^24^ This difference in age results in large differences in CHA_2_DS_2_-VASc-score, in *Table 4*, and clinical risk factors, in Supplementary material online, *Table S6*, adding additional bias to models using risk factors. Using our replication dataset, the 12-lead ECG model with an AUC of 0.84 was outperformed by a simple risk-factor-based model, achieving an AUC of 0.86; age being the main driver of classification, see *Figure 4*. Furthermore, the performance of the 12-lead ECG model can also be explained by age-bias, as Supplementary material online, *Figure S3A* shows the features extracted from the ECG model correlate more strongly with age than with the outcome. This bias lead to unstable model performance, with a large decrease in performance when tested on a matched dataset (*Table 4*).

### Electrocardiogram labelling and selection criteria

Electrocardiograms in SR are assigned to the positive or negative case group based on automatic diagnoses of SR and AF as stored in the MUSE system. An automatic diagnosis of SR is relatively reliable, with a positive predictive value of 93.2% for computer interpretation of ECGs.^33^ These labels are used without correction. AF-labelling is more difficult, with 11.3% being misdiagnosed,^34^ mostly as other arrhythmias. Additionally, an AF diagnosis might have been made at another hospital or in primary care. Both physician corrections to the automatic diagnoses and out-of-hospital diagnoses are frequently present in the patients’ EHR. To reduce the number of false negatives in the negative case group, patients with a structured diagnosis of AF in the EHR system or any likely positive mentioning of AF in their clinical notes were excluded. False positive labels, which can occur in up to 9.3% of cases,^34^ were not corrected for.

The replication cohort was defined as previously described,^14^ but with additional exclusion criteria for data quality. The original study did not exclude same-day SR–ECGs recorded after an AF–ECG treated with cardioversion. To match the study population more closely to a population that would be screened for AF in clinical practice, SR–ECGs recorded after common AF treatments (including cardioversion, see Supplementary material online, *Table S1*) were excluded from the positive case group. In the negative case group, these treatments were used as a proxy for possible missed AF diagnoses in our OMOP dataset and those patients were excluded entirely. This introduces some bias by excluding patients receiving one of these treatments for indications other than AF, especially exclusion based on any use of OAC. However, most (non-AF) indications for these treatments will require direct follow-up, less fitting to the simulated screening scenario. Lastly, ECGs with an AF diagnosis recorded within 7 days from a CABG or heart valve surgery or intervention were excluded from the positive case set. The underlying assumption in detecting AF from SR–ECGs is the presence of some electrocardiographic marker in the ECG due to an underlying electromechanical abnormality, e.g. an atrial myopathy. Coronary artery bypass graft and valve procedures predispose a patient to developing AF, but post-operative AF has a distinct underlying mechanism, with a different ECG signature.^35^

## Supplementary Material

euad354_Supplementary_Data

## Data Availability

Due to the sensitive nature of the health information used, the ECG and OMOP data are not freely available. The AI model architecture is included in Supplementary material online, *File S3*; the trained model is available upon reasonable request.

## References

[1] Roth GA , Mensah GA, Johnson CO, Addolorato G, Ammirati E, Baddour LM et al Global burden of cardiovascular diseases and risk factors, 1990–2019: update from the GBD 2019 study. J Am Coll Cardiol 2020;76:2982–3021. 33309175 10.1016/j.jacc.2020.11.010 PMC7755038

[2] Krijthe BP , Kunst A, Benjamin EJ, Lip GYH, Franco OH, Hofman A et al Projections on the number of individuals with atrial fibrillation in the European Union, from 2000 to 2060. Eur Heart J 2013;34:2746–51. 23900699 10.1093/eurheartj/eht280 PMC3858024

[3] Turakhia MP , Shafrin J, Bognar K, Trocio J, Abdulsattar Y, Wiederkehr D et al Estimated prevalence of undiagnosed atrial fibrillation in the United States. PLoS One 2018;13:e0195088. 29649277 10.1371/journal.pone.0195088 PMC5896911

[4] Savelieva I , Camm AJ. Clinical relevance of silent atrial fibrillation: prevalence, prognosis, quality of life, and management. J Interv Card Electrophysiol 2000;4:369–82. 10936003 10.1023/a:1009823001707

[5] Dilaveris PE , Kennedy HL. Silent atrial fibrillation: epidemiology, diagnosis, and clinical impact. Clin Cardiol 2017;40:413–8. 28273368 10.1002/clc.22667 PMC6490532

[6] Andrade J , Khairy P, Dobrev D, Nattel S. The clinical profile and pathophysiology of atrial fibrillation: relationships among clinical features, epidemiology, and mechanisms. Circ Res 2014;114:1453–68. 24763464 10.1161/CIRCRESAHA.114.303211

[7] Kornej J , Börschel CS, Benjamin EJ, Schnabel RB. Epidemiology of atrial fibrillation in the 21st century: novel methods and new insights. Circ Res 2020;127:4–20. 32716709 10.1161/CIRCRESAHA.120.316340 PMC7577553

[8] Tsao CW , Aday AW, Almarzooq ZI, Anderson CAM, Arora P, Avery CL et al Heart disease and stroke statistics-2023 update: a report from the American Heart Association. Circulation 2023;147:e93–621. 36695182 10.1161/CIR.0000000000001123 PMC12135016

[9] Alonso A , Krijthe BP, Aspelund T, Stepas KA, Pencina MJ, Moser CB et al Simple risk model predicts incidence of atrial fibrillation in a racially and geographically diverse population: the CHARGE-AF consortium. J Am Heart Assoc 2013;2:e000102. 23537808 10.1161/JAHA.112.000102 PMC3647274

[10] Lip GYH , Nieuwlaat R, Pisters R, Lane DA, Crijns HJGM. Refining clinical risk stratification for predicting stroke and thromboembolism in atrial fibrillation using a novel risk factor-based approach: the euro heart survey on atrial fibrillation. Chest 2010;137:263–72. 19762550 10.1378/chest.09-1584

[11] Hindricks G , Potpara T, Dagres N, Arbelo E, Bax JJ, Blomström-Lundqvist C et al 2020 ESC guidelines for the diagnosis and management of atrial fibrillation developed in collaboration with the European Association for Cardio-Thoracic Surgery (EACTS): the task force for the diagnosis and management of atrial fibrillation of the European Society of Cardiology (ESC) developed with the special contribution of the European Heart Rhythm Association (EHRA) of the ESC. Eur Heart J 2021;42:373–498. 32860505 10.1093/eurheartj/ehaa612

[12] Svendsen JH , Diederichsen SZ, Højberg S, Krieger DW, Graff C, Kronborg C et al Implantable loop recorder detection of atrial fibrillation to prevent stroke (the LOOP study): a randomised controlled trial. Lancet 2021;398:1507–16. 34469766 10.1016/S0140-6736(21)01698-6

[13] Svennberg E , Caiani EG, Bruining N, Desteghe L, Han JK, Narayan SM et al The digital journey: 25 years of digital development in electrophysiology from an Europace perspective. Europace 2023;25:euad176. 37622574 10.1093/europace/euad176 PMC10450797

[14] Attia ZI , Noseworthy PA, Lopez-Jimenez F, Asirvatham SJ, Deshmukh AJ, Gersh BJ et al An artificial intelligence-enabled ECG algorithm for the identification of patients with atrial fibrillation during sinus rhythm: a retrospective analysis of outcome prediction. Lancet 2019;394:861–7. 31378392 10.1016/S0140-6736(19)31721-0

[15] Gruwez H , Barthels M, Haemers P, Verbrugge FH, Dhont S, Meekers E et al Detecting paroxysmal atrial fibrillation from an electrocardiogram in sinus rhythm: external validation of the AI approach. JACC Clin Electrophysiol 2023;9:1771–82. 37354171 10.1016/j.jacep.2023.04.008

[16] Noseworthy PA , Attia ZI, Behnken EM, Giblon RE, Bews KA, Liu S et al Artificial intelligence-guided screening for atrial fibrillation using electrocardiogram during sinus rhythm: a prospective non-randomised interventional trial. Lancet 2022;400:1206–12. 36179758 10.1016/S0140-6736(22)01637-3

[17] Lammertyn P-J , Dupulthys S. *Rabbit in a Blender*. https://github.com/RADar-AZDelta/Rabbit-in-a-Blender (9 May 2023, date last accessed).

[18] Khurshid S , Kartoun U, Ashburner JM, Trinquart L, Philippakis A, Khera AV et al Performance of atrial fibrillation risk prediction models in over 4 million individuals. Circ Arrhythm Electrophysiol 2021;14:e008997. 33295794 10.1161/CIRCEP.120.008997 PMC7856013

[19] Quan H , Sundararajan V, Halfon P, Fong A, Burnand B, Luthi J-C et al Coding algorithms for defining comorbidities in ICD-9-CM and ICD-10 administrative data. Med Care 2005;43:1130–9. 16224307 10.1097/01.mlr.0000182534.19832.83

[20] Ribeiro AH , Ribeiro MH, Paixão GMM, Oliveira DM, Gomes PR, Canazart JA et al Automatic diagnosis of the 12-lead ECG using a deep neural network. Nat Commun 2020;11:1760. 32273514 10.1038/s41467-020-15432-4 PMC7145824

[21] Melzi P , Tolosana R, Cecconi A, Sanz-Garcia A, Ortega GJ, Jimenez-Borreguero LJ et al Publisher correction: analyzing artificial intelligence systems for the prediction of atrial fibrillation from sinus-rhythm ECGs including demographics and feature visualization. Sci Rep 2021;11:24030. 34887489 10.1038/s41598-021-03535-x PMC8660788

[22] Lundberg SM , Nair B, Vavilala MS, Horibe M, Eisses MJ, Adams T et al Explainable machine-learning predictions for the prevention of hypoxaemia during surgery. Nat Biomed Eng 2018;2:749–60. 31001455 10.1038/s41551-018-0304-0 PMC6467492

[23] Lundberg SM , Erion G, Chen H, DeGrave A, Prutkin JM, Nair B et al From local explanations to global understanding with explainable AI for trees. Nat Mach Intell 2020;2:56–67. 32607472 10.1038/s42256-019-0138-9 PMC7326367

[24] Noseworthy PA , Attia ZI, Carter RE, Yao X, Friedman PA. An AI-ECG algorithm for atrial fibrillation risk: steps towards clinical implementation—authors’ reply. Lancet 2020;396:236–7. 10.1016/S0140-6736(20)31064-3 32711795

[25] Hygrell T , Viberg F, Dahlberg E, Charlton PH, Kemp Gudmundsdottir K, Mant J et al An artificial intelligence-based model for prediction of atrial fibrillation from single-lead sinus rhythm electrocardiograms facilitating screening. Europace 2023;25:1332–8. 36881777 10.1093/europace/euad036 PMC10105867

[26] Freedman B , Camm J, Calkins H, Healey JS, Rosenqvist M, Wang J et al Screening for atrial fibrillation: a report of the AF-SCREEN international collaboration. Circulation 2017;135:1851–67. 28483832 10.1161/CIRCULATIONAHA.116.026693

[27] Welton NJ , McAleenan A, Thom HH, Davies P, Hollingworth W, Higgins JP et al Screening strategies for atrial fibrillation: a systematic review and cost-effectiveness analysis. Health Technol Assess 2017;21:1–236. 10.3310/hta21290 28629510

[28] Friberg L , Engdahl J, Frykman V, Svennberg E, Levin LÅ, Rosenqvist M. Population screening of 75- and 76-year-old men and women for silent atrial fibrillation (STROKESTOP). Europace 2013;15:135–40. 22791299 10.1093/europace/eus217

[29] Svennberg E , Engdahl J, Al-Khalili F, Friberg L, Frykman V, Rosenqvist M. Mass screening for untreated atrial fibrillation: the STROKESTOP study. Circulation 2015;131:2176–84. 25910800 10.1161/CIRCULATIONAHA.114.014343

[30] Engdahl J , Svennberg E, Friberg L, Al-Khalili F, Frykman V, Kemp Gudmundsdottir K et al Stepwise mass screening for atrial fibrillation using N-terminal pro b-type natriuretic peptide: the STROKESTOP II study design. Europace 2017;19:297–302. 28011798 10.1093/europace/euw319

[31] Gudmundsdottir K , Fredriksson T, Svennberg E, Al-Khalili F, Friberg L, Frykman V et al Stepwise mass screening for atrial fibrillation using N-terminal B-type natriuretic peptide: the STROKESTOP II study. Europace 2020;22:24–32. 31790147 10.1093/europace/euz255 PMC6945054

[32] Attia ZI , Friedman PA, Noseworthy PA, Lopez-Jimenez F, Ladewig DJ, Satam G et al Age and sex estimation using artificial intelligence from standard 12-lead ECGs. Circ Arrhythm Electrophysiol 2019;12:e007284. 31450977 10.1161/CIRCEP.119.007284 PMC7661045

[33] Shah AP , Rubin SA. Errors in the computerized electrocardiogram interpretation of cardiac rhythm. J Electrocardiol 2007;40:385–90. 17531257 10.1016/j.jelectrocard.2007.03.008

[34] Bae MH , Lee JH, Yang DH, Park HS, Cho Y, Chull Chae S et al Erroneous computer electrocardiogram interpretation of atrial fibrillation and its clinical consequences. Clin Cardiol 2012;35:348–53. 22644921 10.1002/clc.22000 PMC6652532

[35] Dobrev D , Aguilar M, Heijman J, Guichard J-B, Nattel S. Postoperative atrial fibrillation: mechanisms, manifestations and management. Nat Rev Cardiol 2019;16:417–36. 30792496 10.1038/s41569-019-0166-5

