# Supplementary material for: Single-lead electrocardiogram Artificial Intelligence model with risk factors detects atrial fibrillation during sinus rhythm
Source: Europace. 2023 Dec 11;26(2):euad354. doi: 10.1093/europace/euad354 (PMC10872711; doi:10.1093/europace/euad354)
Supplement: euad354_Supplementary_Data [file euad354_supplementary_data.zip › supplementary_tables.docx]

Supplementary Table 1: **Selection criteria for ECGs.**
AF=Atrial fibrillation or flutter, CABG=coronary artery bypass graft, LAA=left atrial appendage, ICD=implantable cardioverter-defibrillator, OAC=oral anticoagulation, AAD=antiarrhythmic drugs, EHR=electronic health records.

|  | Both cases | Positive case | Negative case |
| --- | --- | --- | --- |
| **Inclusion** |  |  |  |
| *ECG* | Sinus rhythm | Starting 91 days before first AF | Any point in time |
|  | (age >= 18 years) | (AF not in 7 days following |  |
|  |  | CABG/valve procedure) |  |
| **Exclusion** |  |  |  |
| *ECG* | Poor Quality ECG |  |  |
|  | Paced rhythm detected |  |  |
| *History* | LAA closure | Before ECG | Any point in time |
|  | Ablation therapy | Before ECG | Any point in time |
|  | Cardioversion | Before ECG | Any point in time |
|  | Pacemaker/ICD implant | Before ECG | Any point in time |
|  | Heart transplant | Before ECG | Any point in time |
|  |  |  | Any exposure to OAC |
|  |  |  | Any exposure to AAD |
|  |  |  | Any probable AF in EHR notes |

Supplementary Table 2: **Algorithm hyperparameters for the residual neural networks**. Algorithm hyperparameter tuning was done on an age-prediction task using twelve-lead ECG; results were similar to a previous study[^27^](#ref-Attia2019-fg) for a wide range of algorithm parameters. All other parameters are set to default values in TensorFlow 2.11.1.

| optimizer | Nadam |
| --- | --- |
| learning_rate | 3e-4 |
| beta_1 | 0.9 |
| beta_2 | 0.99 |
| loss | BinaryCrossentropy (with from_logits=True) |
| n_epochs | 50 (with no early stopping) |
| batch_size | 256 |
| validation metric | AUC of the ROC curve |

Supplementary Table 3: **Model hyperparameters for the residual neural networks**. Four models were evaluated, differing only in numbers and types of layers, to compare models with variable complexity and depth. Binary cross-entropy is used as loss function during training. Sample weights used during training are based on the proportion of positive case ECGs over the total number of ECGs in the training set. The optimal set of weights is selected after training as the weights from the epoch with the highest area under the curve of the receiver operating characteristic curve evaluated on the validation dataset, in any of 5 training runs. Model performance is mostly determined by the available input data: one or twelve-lead ECG, with or without risk factors. The model architecture only causes limited changes in model performance; retraining after random initialization has minimal effect. Supplemental figure gives an overview of model performance by architecture. The deep_resnet_encoder architecture gives the best performance overall and is selected for all further analyses. Number of filters and kernel size change gradually over residual units (RUs). Squeeze-excite layers, if any, are in first four RUs. Query, value and key dimensions are equal in encoding units. The number of trainable parameters per model varies slightly, depending on number of ECG-leads (1 or 12) and inclusion of clinical risk factors. RU=Residual Unit, EU=Encoding Unit, S-E=squeeze-excite

|  | shallow_resnet | shallow_resnet_encoder | deep_resnet | **deep_resnet_encoder** |
| --- | --- | --- | --- | --- |
| **residual units** | 6 | 6 | 8 | 8 |
| filters first RU | 8 | 8 | 16 | 16 |
| filters last RU | 64 | 64 | 128 | 128 |
| kernel size first RU | 9 | 9 | 17 | 17 |
| kernel size last RU | 4 | 4 | 8 | 8 |
| RU with S-E layer | 0 | 4 | 0 | 4 |
| **encoding units (EU)** | 0 | 2 | 0 | 2 |
| attention heads in EU |  | 8 |  | 8 |
| key dimension of EU |  | 32 |  | 64 |
| d_model_ of EU |  | 64 |  | 128 |
| d_ff_ of EU |  | 64 |  | 128 |
| **dropout rate** | 0.2 | 0.2 | 0.2 | 0.2 |
| **dense layers** neurons | 64, 32, 16, 8 | 64, 32, 16, 8 | 128, 32, 16, 8 | 128, 32, 16, 8 |
| trainable parameters | ~60.000 | ~200.000 | ~800.000 | ~1.400.000 |

Supplementary Table 4: **Hyperparameter ranges for RandomForestClassifier** from scikit-learn. Hyperparameters are optimized using the same training and validation data as the ResNet. Class weights are the same as the sample weights for the ResNet models. Other parameters left at default values.

| n_estimators | 10, 30, 100 |
| --- | --- |
| criterion | entropy, log_loss |
| min_samples_leaf | 10, 30, 100 |

Supplementary Table 5: **Study population characteristics for matched dataset** shows reduced differences by age-filtering, by using a smaller time window around the first AF-ECG and especially by age- (and sex)-matching the negative cases. The remaining differences between positive and negative cases should mainly be related to the atrial fibrillation risk. Age and CHA_2_DS_2_-VASc-score as ‘median (first quartile-third quartile)’. +=positive case, –=negative case, val=validation

|  | **+** (train) | **–⁠** (train) | **+** (val/test) | **–⁠** (val/test) |
| --- | --- | --- | --- | --- |
| Age (years) | 75 (67—82) | 73 (66—81) | 75 (68—83) | 74 (66—82) |
| Sex (male) | 60.76% | 60.76% | 58.44% | 58.44% |
| CHA_2_DS_2_-VASc-score | 3 (2—4) | 3 (1—4) | 3 (2—4) | 2 (1—3) |
| Obesity or BMI >= 30 | 19.27% | 14.65% | 18.85% | 13.44% |
| Smoking (any exposure) | 30.87% | 24.04% | 25.98% | 19.57% |
| Coronary artery bypass graft present | 18.68% | 10.78% | 13.11% | 6.17% |
| Chronic kidney disease | 23.35% | 11.61% | 19.59% | 9.32% |
| Chronic obstructive pulmonary disease | 18.49% | 10.53% | 14.59% | 8.20% |
| Diabetes mellitus | 24.26% | 17.95% | 21.72% | 14.75% |
| Heart failure | 23.10% | 7.01% | 20.08% | 4.55% |
| Heart valve disease or valve procedure | 19.57% | 6.10% | 15.66% | 3.61% |
| Hypertension | 50.51% | 33.59% | 45.49% | 27.36% |
| Hyperthyroidism | 2.72% | 1.25% | 2.13% | 1.17% |
| Hypothyroidism | 5.34% | 3.01% | 3.52% | 2.52% |
| Myocardial infarction | 46.00% | 27.81% | 37.70% | 21.50% |
| Obstructive sleep apnoea syndrome | 3.41% | 2.22% | 2.21% | 2.25% |
| Peripheral artery disease | 14.32% | 7.26% | 9.10% | 5.72% |
| Stroke or transient ischaemic attack | 10.85% | 8.63% | 10.33% | 7.91% |

Supplementary Table 6: **Study population characteristics for replication dataset** showing stark differences for negative cases between the training and validation/test set. Differences between positive and negative cases are related to atrial fibrillation risk, but exaggerated by age-bias in ECG-selection strategies. Age and CHA_2_DS_2_-VASc-score as ‘median (first quartile-third quartile)’. +=positive case, –=negative case, val=validation

|  | **+** (train) | **–⁠** (train) | **+** (val/test) | **–⁠** (val/test) |
| --- | --- | --- | --- | --- |
| Age (years) | 75 (67—82) | 62 (50—74) | 76 (68—83) | 57 (44—70) |
| Sex (male) | 60.76% | 46.26% | 58.44% | 46.05% |
| CHA_2_DS_2_-VASc-score | 4 (2—5) | 2 (1—3) | 3 (2—5) | 1 (0—2) |
| Obesity or BMI >= 30 | 21.25% | 13.51% | 20.42% | 8.85% |
| Smoking (any exposure) | 32.46% | 20.44% | 28.73% | 11.64% |
| Coronary artery bypass graft present | 25.51% | 6.61% | 18.15% | 1.07% |
| Chronic kidney disease | 26.60% | 7.14% | 21.36% | 2.48% |
| Chronic obstructive pulmonary disease | 19.39% | 7.04% | 16.82% | 3.52% |
| Diabetes mellitus | 25.54% | 12.69% | 24.01% | 6.62% |
| Heart failure | 26.30% | 4.58% | 24.20% | 1.20% |
| Heart valve disease or valve procedure | 22.39% | 3.88% | 24.39% | 1.00% |
| Hypertension | 56.25% | 23.91% | 49.53% | 10.55% |
| Hyperthyroidism | 4.51% | 0.96% | 2.84% | 0.43% |
| Hypothyroidism | 7.05% | 2.45% | 4.91% | 1.07% |
| Myocardial infarction | 54.10% | 21.02% | 42.16% | 9.71% |
| Obstructive sleep apnoea syndrome | 4.33% | 2.29% | 3.40% | 1.43% |
| Peripheral artery disease | 15.32% | 4.34% | 11.34% | 1.78% |
| Stroke or transient ischaemic attack | 12.91% | 5.73% | 15.69% | 2.88% |
