# Supplementary material for: Single-lead electrocardiogram Artificial Intelligence model with risk factors detects atrial fibrillation during sinus rhythm
Source: Europace. 2023 Dec 11;26(2):euad354. doi: 10.1093/europace/euad354 (PMC10872711; doi:10.1093/europace/euad354)
Supplement: euad354_Supplementary_Data [file euad354_supplementary_data.zip › supplementary_figures.docx]

| Matched dataset | Replication dataset |
| --- | --- |
| A 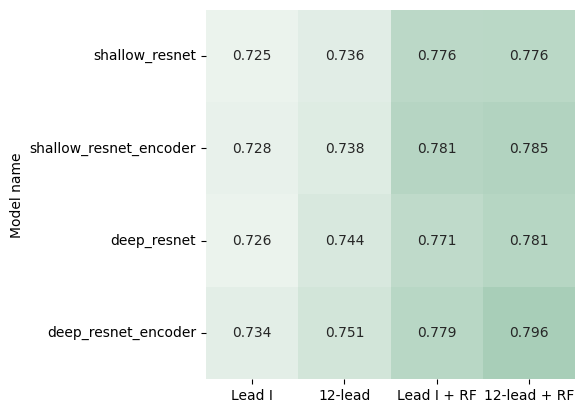 | B 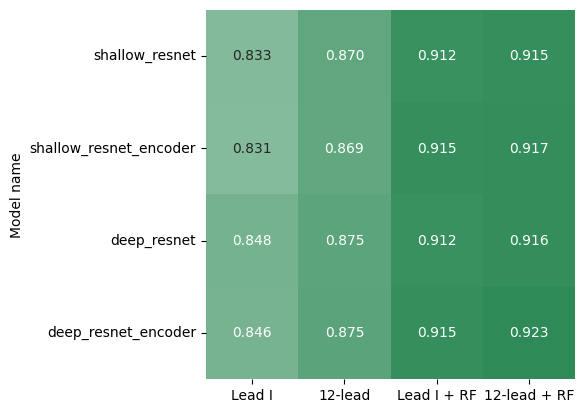 |

Supplementary Figure 1: **Area under the receiver operating characteristic curve of all four ResNet architectures** trained on the training set and evaluated on the validation set for A, the matched dataset and B, the replication dataset. Performance depends mostly on the input data; the architecture has limited effect, but overall, the larger models perform slightly better, so the deep_resnet_encoder architecture is used for all analyses.


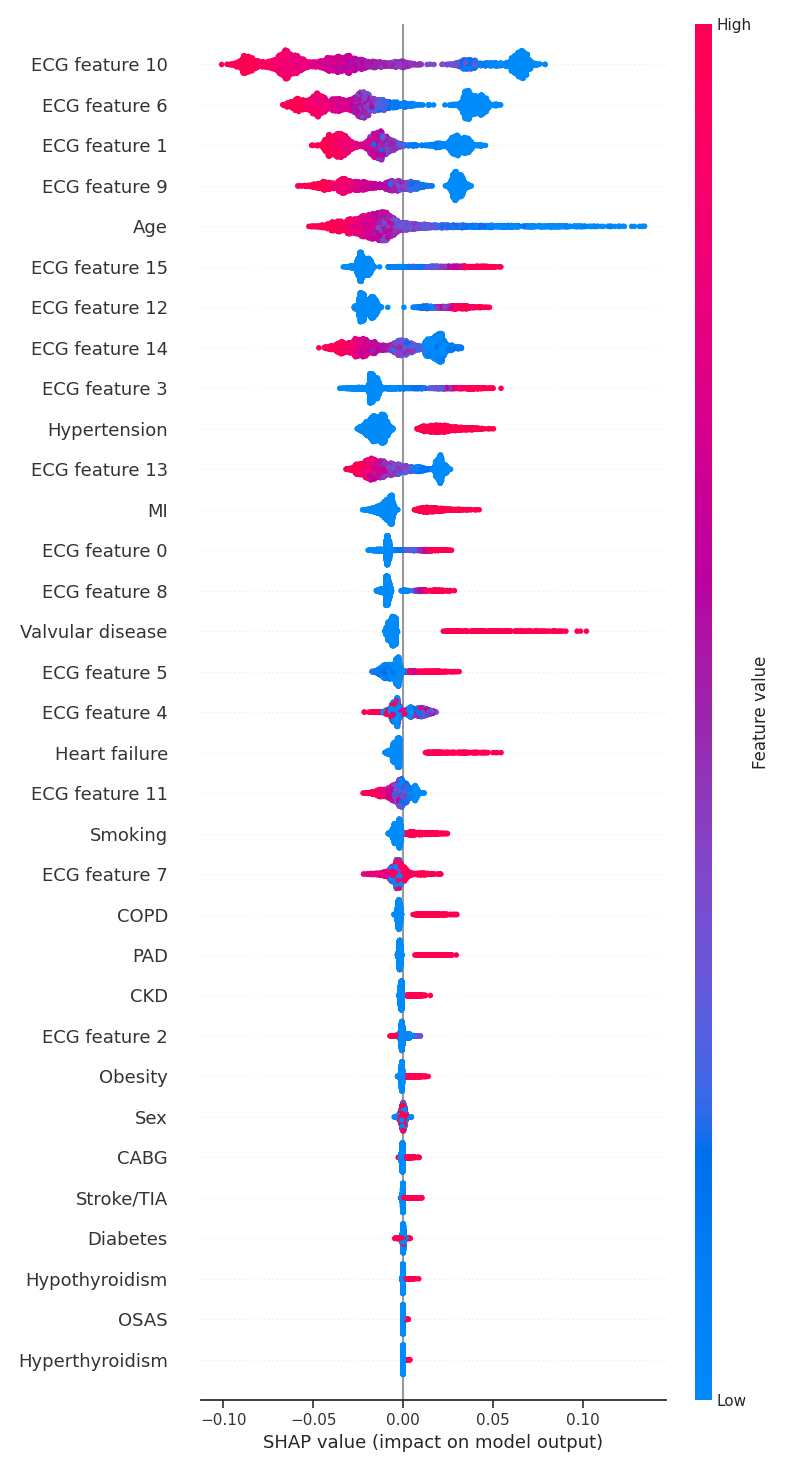


Supplementary Figure 2: **Shap values for the Random Forest classifier using risk factors and an ECG embedding vector** for the matched dataset. ECG features and cardiovascular diseases dominate. AUC=area under the curve

| A 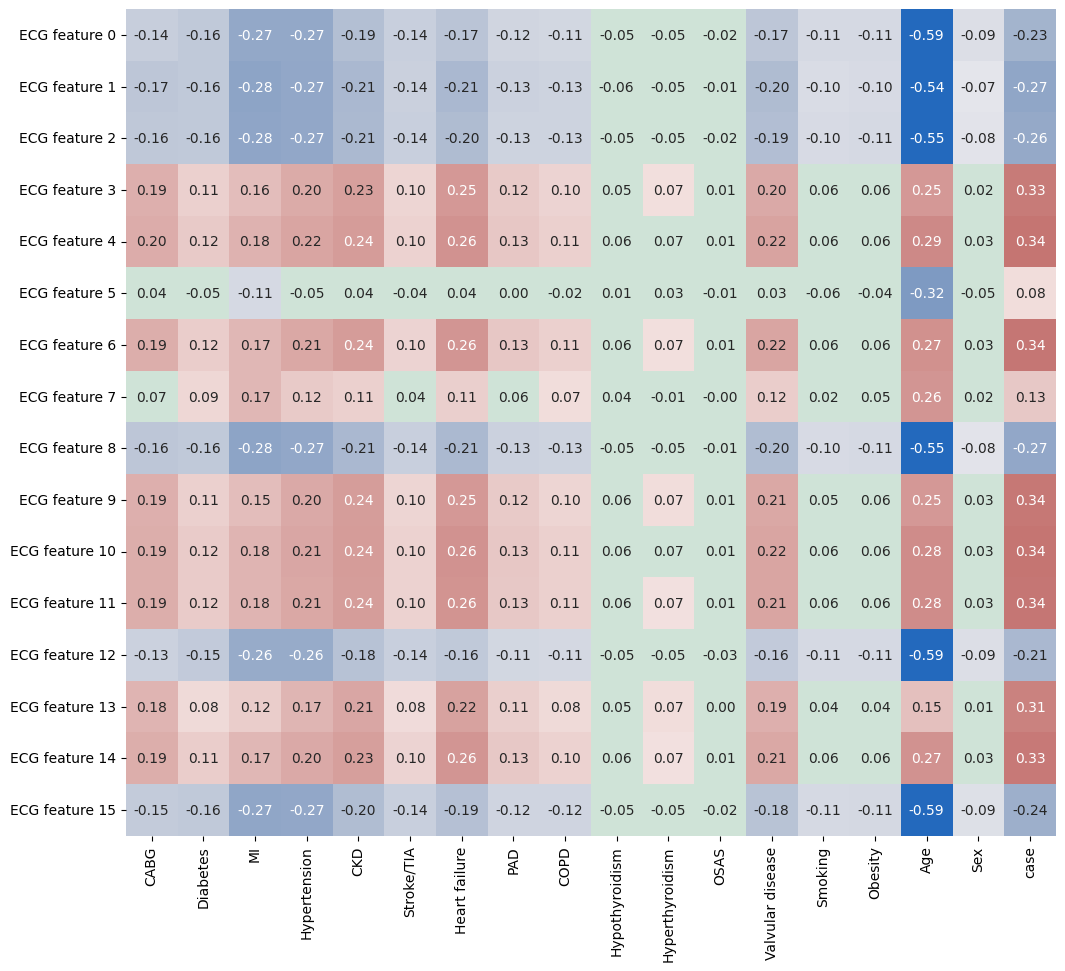 | B 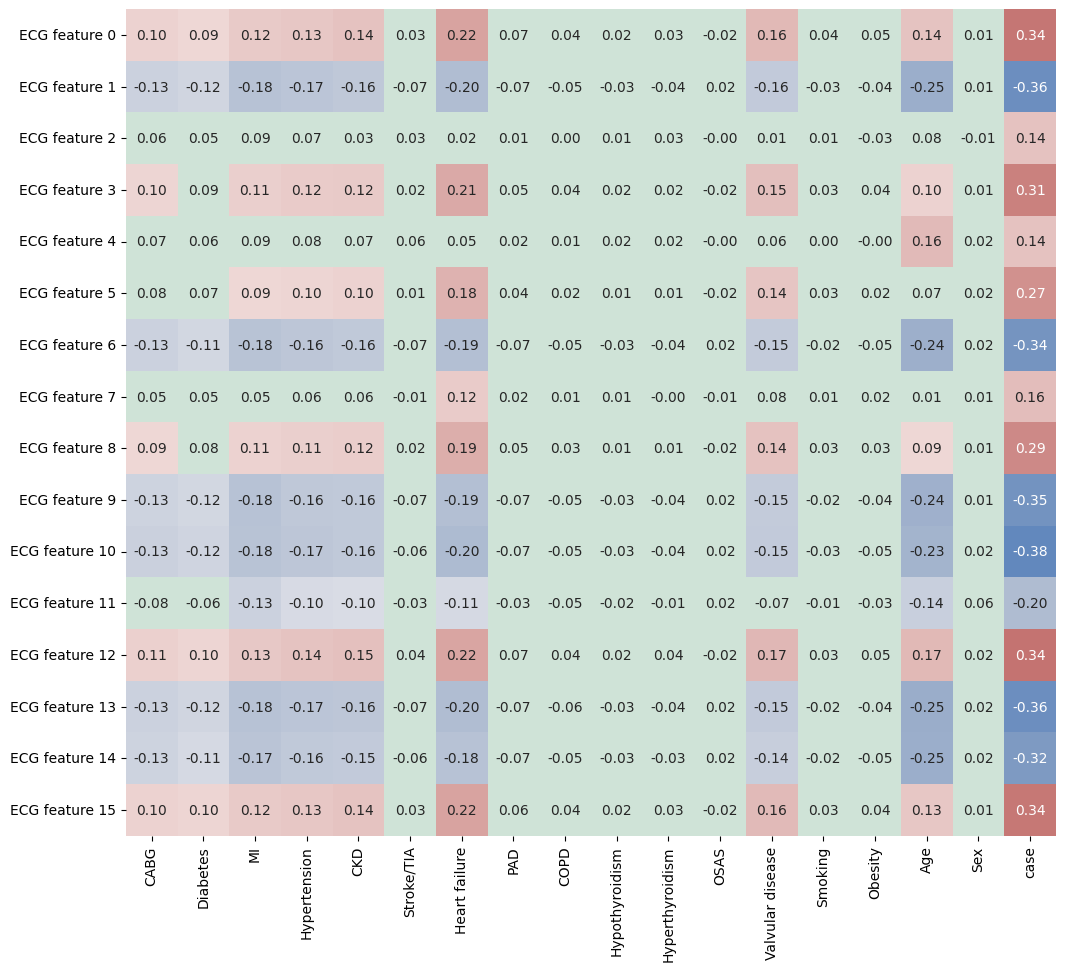 |
| --- | --- |

Supplementary Figure 3: A, **Correlation for the replication dataset between risk factors and an ECG feature vector** from the 12-lead ECG model, showing stronger correlation with age than with the outcome as a positive or negative case). B, **Correlation for the matched dataset**, where the strongest correlations are now between the ECG features and the predicted outcome. Insignificant values in green (i.e., Bonferroni-corrected p-value > 0.05). MI=myocardial infarction, CABG=coronary artery bypass graft, CKD=chronic kidney disease, PAD=peripheral artery disease, COPD=chronic obstructive pulmonary disease, TIA=transient ischaemic attack, OSAS=obstructive sleep apnoea syndrome


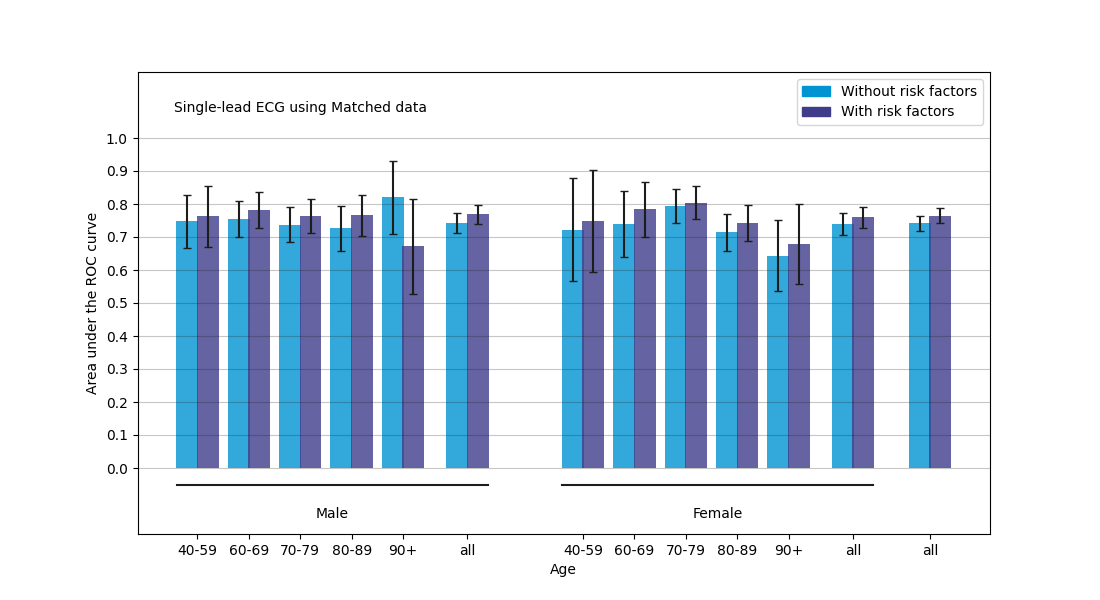
Supplementary Figure 4: **Age- and sex-stratified AUC** for single-lead ECG model with and without (all) risk factors for the matched dataset. A small, but consistent, benefit is seen from adding risk factors to the model, except for men over 90 years, possibly due to very small sample size (7 ECGs in the test set).
